# First large-scale genomic prediction in the honey bee

**DOI:** 10.1038/s41437-023-00606-9

**Published:** 2023-03-06

**Authors:** Richard Bernstein, Manuel Du, Zhipei G. Du, Anja S. Strauss, Andreas Hoppe, Kaspar Bienefeld

**Affiliations:** Institute for Bee Research Hohen Neuendorf, Friedrich-Engels-Str. 32, 16540 Hohen Neuendorf, Germany

**Keywords:** Agriculture, Genomics

## Abstract

Genomic selection has increased genetic gain in several livestock species, but due to the complicated genetics and reproduction biology not yet in honey bees. Recently, 2970 queens were genotyped to gather a reference population. For the application of genomic selection in honey bees, this study analyzes the accuracy and bias of pedigree-based and genomic breeding values for honey yield, three workability traits, and two traits for resistance against the parasite *Varroa destructor*. For breeding value estimation, we use a honey bee-specific model with maternal and direct effects, to account for the contributions of the workers and the queen of a colony to the phenotypes. We conducted a validation for the last generation and a five-fold cross-validation. In the validation for the last generation, the accuracy of pedigree-based estimated breeding values was 0.12 for honey yield, and ranged from 0.42 to 0.61 for the workability traits. The inclusion of genomic marker data improved these accuracies to 0.23 for honey yield, and a range from 0.44 to 0.65 for the workability traits. The inclusion of genomic data did not improve the accuracy of the disease-related traits. Traits with high heritability for maternal effects compared to the heritability for direct effects showed the most promising results. For all traits except the *Varroa* resistance traits, the bias with genomic methods was on a similar level compared to the bias with pedigree-based BLUP. The results show that genomic selection can successfully be applied to honey bees.

## Introduction

Genomic selection (Meuwissen et al. 2001) incorporates genome-wide marker data into breeding value estimation. Compared to pedigree-based breeding values, the use of genomic data can increase the accuracy of estimated breeding values (EBV), or enable the selection of animals before they are phenotyped. Both strategies have been realized to increase the genetic gain in several livestock species (Doublet et al. 2019; Fulton 2012; Samorè and Fontanesi 2016). Honey bee breeders, by contrast, employ phenotypic selection (De la Mora et al. 2020; Maucourt et al. 2020) or pedigree-based breeding value estimation (Bienefeld et al. 2007; Brascamp et al. 2016; Hoppe et al. 2020). Recently, a high-density SNP chip was developed and genotypes of phenotyped queens are now available to validate genomic prediction (Jones et al. 2020).

Pedigree-based best linear unbiased prediction (PBLUP) of breeding values began in 1994 for the population registered on BeeBreed. The EBV enabled hundreds of mostly Central European bee breeders to improve the quality of their stock (Hoppe et al. 2020). To ensure the quality of the EBV, the program relies on a specialized infrastructure for mating control and an adapted genetic model to account for the peculiarities of the honey bee (Bienefeld et al. 2007; Brascamp and Bijma 2014).

The phenotypes of honey bee colonies for economically relevant traits result from the collaboration of worker groups and queens. In honey yield, for example, the workers of a colony perform foraging and storing, but the queen affects the number of workers via her egg-laying rate, and influences the behavior of the workers via pheromones. Therefore, the genetic model for the traits includes direct and maternal effects for the contribution of workers and queens, respectively.

In commercial honey bee breeding programs, the demands of beekeepers lead to selection traits that differ significantly in terms of methodology and effort for recording and mathematical modelling. Typical aims include increased honey yield, better workability for the beekeeper, and more disease resistance (Petersen et al. 2020; Uzunov et al. 2017). Especially resistance against *Varroa destructor* is targeted, since this parasitic mite contributes to severe colony losses in numerous countries (Genersch et al. 2010; Guichard et al. 2020; Traynor et al. 2016).

Genomic breeding value estimation in honey bees has been tried in simulation studies, and single-step genomic BLUP (ssGBLUP) appeared as an efficient solution (Bernstein et al. 2021; Gupta et al. 2013) to combine pedigree information with genomic information. The simulations showed that ssGBLUP can increase the accuracy of genomic breeding values considerably and enables high genetic gains, if the infrastructure is appropriately adapted. Augmenting ssGBLUP with trait-specific weights leads to weighted ssGBLUP (WssGBLUP) (Wang et al. 2012), which can increase the prediction accuracy further, as results from other species have shown (Lourenco et al. 2014; Teissier et al. 2019; Vallejo et al. 2019).

To our knowledge, only simulated results on genomic EBV in honey bees have been published until now. In this study, we first report the accuracies and the bias of PBLUP, ssGBLUP, and WssGBLUP for a number of key traits of economic importance in a large breeding population of honey bees.

## Materials and methods

### Data

Pedigree and performance data from the *Apis mellifera carnica* population were used, since the genotyped queens belonged to this subspecies, which is native and widespread in Central Europe (Lodesani and Costa 2003; Ruttner 1988; Wallberg et al. 2014). The data were downloaded from BeeBreed in February 14, 2021, totaling 201,304 valid performance tests and pedigree data of 234,519 queens. The oldest queen on the pedigree was born in 1949. Since a large part of the BeeBreed data set was of negligible relevance to the breeding values of the genotyped queens, the data were reduced and refined for the comparison of classical and genomic prediction. Queens with a valid phenotype whose genotypes passed the quality control (see below) were the starting set. In an iterative process, phenotypes of performance-tested queens on apiaries from the test year 2010 onwards were included by adding (1) queens tested at the apiaries of the previously added queens, (2) sister queens of the previously added queens, and (3) queens when an ancestor as well as offspring had already been added. Steps (1)–(3) were repeated until no further phenotypes could be added. The pedigree was restricted to the resulting queens and their ancestors. The final enriched data set contained 36,509 phenotypes in a pedigree of 44,183 queens and 4512 sires, which were usually groups of sister queens dedicated to drone production in an isolated geographic area. Table 1 lists the countries of origin for all colonies.

**Table 1:** Number of phenotyped and genotyped queens included in the data set by the country.

Country	Phenotyped queens	Genotyped queens after quality control
Germany	24,019	1982
Austria	9618	372
Italy	796	1
Switzerland	619	17
Ukraine	467	0
Belgium	368	4
The Netherlands	275	11
Sweden	133	0
France	117	0
Croatia	91	2
Total	36,503	2389

For 6 queens in the data set, no country of origin was given, and they were not genotyped.

The phenotypes covered honey yield, gentleness, calmness, swarming drive, hygienic behavior, and *Varroa* infestation development (VID). Honey yield was measured in kg, and the values were corrected for outliers as described in (Hoppe et al. 2020). Gentleness, calmness, and swarming tendency were recorded as marks from 1 to 4 with 4 being the best mark. Records for these traits were discarded if all colonies on an apiary received the same mark. For hygienic behavior, larvae were artificially killed with a pin and the percentage of cleared cells was recorded (Büchler et al. 2013). VID indicates the resistance of a colony against *Varroa*, based on the change in the level of *Varroa* infestation from early spring to late summer (see Hoppe et al. 2020 for the calculation of VID). For a measurement of *Varroa* infestation, a bee sample is taken from the hive, and the number of mites per 10 g bees is determined (Büchler et al. 2013). Table 2 shows the descriptive statistics of the phenotypes available for each trait.

**Table 2:** Descriptive statistics for honey yield, gentleness, calmness, swarming drive, hygienic behavior, and *Varroa* infestation development (VID).

Trait	Number of records	Number of genotyped queens with record	Average size of apiaries with a genotyped queen (SE)	Mean	SD	Min.	Max.
Honey yield	35,888	2046	13.62 (8.33)	40.71	22.84	0	199.8
Gentleness	35,187	2013	13.80 (8.48)	3.52	0.48	1	4
Calmness	34,652	2016	13.76 (8.50)	3.49	0.48	1	4
Swarming drive	26,937	1549	14.57 (8.88)	3.55	0.76	1	4
Hygienic behavior	23,924	1781	13.36 (7.86)	62.26	23.13	0	100
VID	24,650	1787	13.48 (7.85)	−1.55	2.38	−77.12	6.93

Honey yield is given in kg. Marks from 1 to 4 were recorded for gentleness, calmness, and swarming drive. Hygiene is given as the percentage of cleared cells. VID is a *Varroa* resistance score and higher values indicate more resistance.

The 100-K-SNP chip (Jones et al. 2020) was used to genotype 2970 queens which were registered on BeeBreed and born between 2009 and 2017. Markers that were called in less than 90% of the samples, had minor allele frequency below 1%, or showed significant deviations from Hardy–Weinberg equilibrium after Bonferroni-correction (*χ*^2^ *p* value < 0.05 × 10^–5^) were removed. This left 63,240 markers for further analysis. A total of 312 queens were removed because less than 90% of all the valid markers were called in their samples, indicating low DNA quality. After comparisons of daughter and parent based on the number of opposing homozygotes, 207 queens were removed (Bernstein et al. 2022). Subsequently, 62 samples were removed based on the comparison of genomic and classic relationship matrix (Calus et al. 2011). This left 2389 genotyped queens for further analysis.

### Model and genetic parameters

The complex collaboration between the workers and the queen of a colony must be reflected in the model, and carefully analyzed in the calculation of genetic parameters (Brascamp and Bijma 2019). The phenotype, *y*, of a colony is modelled as follows:

1 $$y = a_W + m_Q + e$$

where *a*_*w*_ is the direct effect of the worker group in the colony, and *m*_*Q*_ the maternal effect of the queen in the colony, while *e* is a non-heritable residual. The genetic component of the phenotype will be denoted *g* = *a*_*W*_ + *m*_*Q*_.

The phenotypic variance was calculated according to formula (2) in Brascamp and Bijma (2019) as follows:

2 $$\sigma _{ph}^2 = A_{base}\sigma _a^2 + \sigma _m^2 + \sigma _{am} + \sigma _e^2$$

where $\sigma _a^2$ and $\sigma _m^2$ are the additive genetic variances of direct and maternal effects, *σ*_*am*_ is the covariance between direct and maternal effects, $\sigma _a^2$ is the residual variance, and *A*_*base*_ is the average relationship between two workers of the same colony in the base population. The variance components were estimated via AIREML with the complete phenotypic information, using the model for PBLUP (see below). We used *A*_*base*_ = 0.40 (Brascamp and Bijma 2019), because even the oldest queens in our pedigree came from populations with established mating control (Armbruster 1919). The heritabilities of direct and maternal effects, $h_a^2$ and $h_m^2$ were calculated according to formulas (6b) and (6c) in Brascamp and Bijma (2019), respectively, as follows:

3 $$h_a^2 = A_{base}\sigma _a^2/\sigma _{ph}^2\,{\rm{and}}\,h_{m}^{2} = \sigma _{m}^{2}/\sigma_{ph}^{2}$$

We provide two concepts of the heritability of the sum of maternal and direct effects. Firstly, heritability is usually defined as the fraction of phenotypic variance due to additive genetic effects. In honey bees, the corresponding concept is the heritability of the genetic component of the phenotype, $h_g^2 = {{{\mathrm{Var}}}}\left( g \right)/\sigma _{ph}^2$ . We calculate $h_g^2$ according to formula (6a) in Brascamp and Bijma (2019) as follows:

4 $$h_g^2 = \frac{{A_{base}\sigma _a^2 + \sigma _m^2 + \sigma _{am}}}{{\sigma _{ph}^2}}$$

Secondly, in the classical theory of animal breeding, the heritability can be used to predict short-term genetic gain, but $h_g^2$ is unsuitable for this purpose. The BeeBreed data set relies on colony-based selection (CBS), and short-term genetic gain with CBS can be estimated using formulas (18) and (6) from Bernstein et al. (2021) using the heritability of the selection criterion of CBS, $h_{CBS}^2$. We calculate $h_{CBS}^2$ as follows:

5 $$h_{CBS}^2 = A_{base}\frac{{\sigma _a^2 + \sigma _m^2 + 2\sigma _{am}}}{{\sigma _{ph}^2}}$$

The numerators of $h_g^2$ and $h_{CBS}^2$ correspond to the notions of genetic variance in the performance and selection criterion, respectively, as introduced by Du et al. (2021).

### Breeding value estimation

We analyzed single-trait models without repeated measurements for the same trait on the same colony. The following mixed linear model was used for PBLUP:

6 $${{{\mathbf{y}}}} = {{{\mathbf{Xb}}}} + {{{\mathbf{Z}}}}_a{{{\mathbf{a}}}} + {{{\mathbf{Z}}}}_m{{{\mathbf{m}}}} + {{{\mathbf{e}}}}$$

where **y** is a vector of observations on colonies; **b** a vector of fixed effects (year and apiary); **a** a vector of direct effects of queens, worker groups or sires; **m** a vector of maternal effects of queens, worker groups or sires; **e** a vector of residuals; and **X**, **Z**_*a*_, and **Z**_*m*_ are known incidence matrices for **b**, **a**, and **m**, respectively. For **a**, **m**, and **e**, the expected values were assumed to equal **0**, while their covariance matrix was given by:

7 $${{{\mathrm{Var}}}}\left( {\begin{array}{*{20}{c}} {{{\mathbf{a}}}} \\ {{{\mathbf{m}}}} \\ {{{\mathbf{e}}}} \end{array}} \right) = \left( {\begin{array}{*{20}{c}} {\sigma _a^2{{{\mathbf{A}}}}} & {\sigma _{am}{{{\mathbf{A}}}}} & 0 \\ {\sigma _{am}{{{\mathbf{A}}}}} & {\sigma _m^2{{{\mathbf{A}}}}} & 0 \\ 0 & 0 & {\sigma _e^2{{{\mathbf{I}}}}} \end{array}} \right)$$

where **A** is the honey bee-specific numerator relationship matrix derived from pedigree (Brascamp and Bijma 2014), **I** is an identity matrix, and $\sigma _a^2$, $\sigma _m^2$, $\sigma _{am}$ and $\sigma _e^2$ are the additive genetic variance of worker and queen effects, their covariance, and the residual variance, respectively.

The model equation and variances for ssGBLUP were the same as for PBLUP, except for the fact that matrix **H** replaced matrix **A**. Matrix **H** was constructed from the numerator relationship matrix **A** which is calculated from pedigree information, and the marker information in the following steps (Aguilar et al. 2010; Christensen and Lund 2010). The genomic relationship matrix, **G**, (VanRaden 2008, method 1) was constructed by the following equation:

8 $${{{\mathbf{G}}}} = \frac{{{{{\mathbf{ZZ}}}}^T}}{{2\mathop {\sum}\nolimits_i {p_i\left( {1 - p_i} \right)} }}$$

where *p*_*i*_ is the allele frequency of the SNP at locus *i*; **Z** = **M**–**P** with **M** containing the marker information of all genotyped queens given as 0, 1, 2, and matrix **P** defined column-wise by *P*_*ji*_ = 2*p*_*i*_ for all *j*. Matrix **G** was adjusted to **A** by adjusting the means of diagonal and off-diagonal elements as described by (Christensen et al. 2012). To have an invertible genomic relationship matrix, we used the weighted genomic relationship matrix, **G**_*w*_, given by the following equation:

9 $${{{\mathbf{G}}}}_w = 0.95{{{\mathbf{G}}}} + 0.05{{{\mathbf{A}}}}_g$$

where **A**_*g*_ is the submatrix of **A** relating to the genotyped animals. Finally, the inverse of **H** was computed according to the following formula:

10 $${{{\mathbf{H}}}}^{ - 1} = {{{\mathbf{A}}}}^{ - 1} + \left( {\begin{array}{*{20}{c}} 0 & 0 \\ 0 & {{{{\mathbf{G}}}}_w^{ - 1} - {{{\mathbf{A}}}}_g^{ - 1}} \end{array}} \right)$$

Method WssGBLUP is an expansion of ssGBLUP which employs weights for all marker loci in the construction of the numerator relationship matrix. In order to assign a large weight to loci with a high impact on the trait, the weight of a single marker locus corresponds to the amount of additive genetic variance explained by this locus. To calculate the additive genetic variance explained by each marker, a BLUP equation for the SNP effects was used.

The model equation and variances for WssGBLUP were the same as for ssGBLUP, except for the fact that matrix G^*^ replaced matrix **G**. Matrix G^*^ was constructed from the vectors of direct and maternal additive genetic effects, **a** and **m**, and the genomic relationship matrix **G**_*w*_, which were obtained from ssGBLUP. The vectors of the direct and maternal SNP effects, **u** and **v**, were estimated by:

11 $$\begin{array}{l}{\mathbf{u}}=\lambda {{\mathbf{M}}}^{T} {\mathbf{G}}^{-1}_{w}{\mathbf{a}}\\{{{\mathbf{v}}}} = \lambda {{{\mathbf{M}}}}^T{{{\mathbf{G}}}}_w^{ - 1}{{{\mathbf{m}}}}\end{array}$$

with $\lambda = \frac{1}{{2\mathop {\sum}\nolimits_i {p_i\left( {1 - p_i} \right)} }}$, where *p*_*i*_ and **M** have the same value as in ssGBLUP. SNP weights **d** were calculated using the average of the direct and maternal SNP effects, deviating from the original algorithm which considered only single-trait models (Wang et al. 2012) as follows:

12 $$d_i = \left( {\frac{{u_i + v_i}}{2}} \right)^22p_i\left( {1 - p_i} \right)$$

Diagonal matrix **D** was defined by $D_{ii} = d_i/\overline {{{\mathbf{d}}}}$, where $\overline {{{\mathbf{d}}}}$ is the average of **d**. The trait-specific matrix G^*^ was calculated by the following formula:

13 $${{{\mathbf{G}}}}^ \ast = \frac{{{{{\mathbf{ZDZ}}}}^T}}{{2\mathop {\sum}\nolimits_i {p_i\left( {1 - p_i} \right)} }}$$

where **Z** is the same matrix as in ssGBLUP.

Programs from the BLUPF90 software (Misztal et al. 2002) were used to estimate the genetic parameters, predict breeding values and calculate relationship matrices **G** and G^*^. To account for the specifics of honey bees, PInCo (Bernstein et al. 2018) was used to calculate the pedigree-based relationship matrices. Equations (9)–(12) were implemented in R (R Development Core Team 2020).

### Validation

We performed two types of cross-validation. The generation validation simulated the selection of candidates before they were phenotyped, which is a common scenario in genomic selection. However, the differences in management practices, climate, and vegetation between apiaries can influence the results of the generation validation. The five-fold cross-validation was designed to evaluate predicted breeding values with a reduced impact of the differences between apiaries.

In the generation validation, EBV were predicted using PBLUP, ssGBLUP and WssGBLUP (1) without the phenotypes of all queens born in 2017 or later, and (2) without the phenotypes of queens born in 2016 or later. For the validation procedure, the EBV of the 265 genotyped queens born in 2017 from scenario 1 were merged with the EBV of the 994 genotyped queens born in 2016 from scenario 2, and likewise for the EBV of the corresponding worker groups. Thereby, the validation sets of the two scenarios, i.e., the genotyped queens born in 2017 and 2016, respectively, could be treated as a single validation set. In the five-fold cross-validation, only apiaries with at least five performance-tested queens were included to ensure reliable estimates of fixed effects. This left 1281 genotyped queens for validation. Each apiary was randomly split into five equally sized partitions, splitting the 1281 queens into five partitions. For each partition, EBV were estimated using PBLUP, ssGBLUP and WssGBLUP without the phenotypes of the animals on this partition. The results from all partitions were merged, so that the five partitions could be treated as a single validation set of 1281 queens and their worker groups. The procedure was repeated six times from the split of the apiaries on.

To assess the accuracy of PBLUP, ssGBLUP, and WssGBLUP, we calculated the accuracy of the prediction of the genetic component of the phenotype, *g*, as follows:

14 $$r_{{{{\boldsymbol{g}}}},{{{\hat{\boldsymbol g}}}}} = \frac{{r_{{{{\boldsymbol{y}}}} - {{{\boldsymbol{Xb}}}},\widehat {{{\boldsymbol{g}}}}}}}{{h_g}}$$

where $\widehat {{{\boldsymbol{g}}}}$ was calculated for each colony, *C*, by $\widehat g_C = \widehat a_W + \widehat m_Q$ with $\widehat a_W$ as the predicted direct effect of the worker group of *C*, $\widehat m_Q$ as the predicted maternal effect of the queen of *C*, and y − ***Xb*** as the vector of phenotypes corrected for fixed effects. We prove Eq. (14) in the Appendix (Text S1). For each method to predict EBV, the phenotypes corrected for fixed effects were calculated using fixed effects from the same method. In the generation validation, PBLUP, ssGBLUP and WssGBLUP were run on the complete data set to obtain appropriate fixed effects. In the five-fold cross-validation, the fixed effects for the correction of the phenotypes were taken from the same run of the same partition as the predicted phenotypes.

A bootstrap procedure was used to test whether the accuracies of WssGBLUP and ssGLUP were significantly higher than the accuracy of PBLUP. In total, 10,000 bootstrap sample vectors were constructed by sampling validation queens with replacement, and the accuracy with PBLUP, ssGLUP, and WssGBLUP was calculated for each vector. Two methods were considered significantly different, if the same method had higher accuracy in 97.5% of all sample vectors (*p* value of 0.05 in a two-sided test). Similar bootstrapping methods were used in other studies (Iversen et al. 2019; Legarra et al. 2008).

The regression coefficient, *b*_1_, of ***y*** − ***Xb*** on $\widehat {{{\boldsymbol{g}}}}$ was used as a measure of bias. Values of *b*_1_ < 1 and *b*_1_ > 1 indicate inflation and deflation of the genetic components of the phenotypes compared to the phenotypes corrected for fixed effects, respectively.

## Results

### Genetic parameters

Estimates of the genetic parameters are shown in Table 3. The heritability of the genetic component of the phenotype, $h_g^2$, was very high for gentleness and calmness, medium for hygienic behavior, honey yield and swarming drive, low for VID. All traits showed considerable negative genetic correlations between maternal and direct effects. The heritability for direct effects was considerably larger than the heritability for maternal effects in gentleness, calmness, and hygienic behavior, but equal to or smaller than the heritability for maternal effects for all other traits.

**Table 3:** Estimated variance and covariance components, genetic parameters derived from these (co)variances.

Trait	$\sigma _a^2$	$\sigma _m^2$	*σ*_*am*_	$\sigma _e^2$	$h_a^2$	$h_m^2$	*r*_*G*_	$h_g^2$	$h_{CBS}^2$
Honey yield	27.235 (5.406)	13.841 (3.015)	−6.124 (3.372)	61.549 (1.476)	0.136 (0.027)	0.173 (0.037)	−0.315 (0.142)	0.232 (0.02)	0.144 (0.019)
Gentleness	0.134 (0.016)	0.027 (0.006)	−0.020 (0.008)	0.062 (0.003)	0.435 (0.053)	0.221 (0.049)	−0.325 (0.096)	0.497 (0.029)	0.396 (0.033)
Calmness	0.103 (0.013)	0.019 (0.005)	−0.013 (0.006)	0.059 (0.003)	0.387 (0.048)	0.181 (0.043)	−0.289 (0.105)	0.448 (0.028)	0.363 (0.03)
Swarming drive	0.143 (0.033)	0.054 (0.017)	−0.008 (0.019)	0.356 (0.010)	0.124 (0.029)	0.117 (0.037)	−0.087 (0.249)	0.224 (0.024)	0.158 (0.022)
Hygienic behavior	111.61 (22.027)	25.571 (8.508)	−5.454 (10.688)	174.73 (5.688)	0.186 (0.037)	0.107 (0.035)	−0.102 (1.645)	0.270 (0.026)	0.211 (0.026)
VID	0.159 (0.045)	0.068 (0.024)	−0.028 (0.027)	0.619 (0.014)	0.088 (0.025)	0.095 (0.033)	−0.270 (0.275)	0.144 (0.02)	0.095 (0.018)

The approximate standard errors are given in brackets.

VID, *Varroa* infestation development; the last nine columns show the additive genetic variances of direct ($\sigma _a^2$) and maternal effects ($\sigma _m^2$), their covariance (*σ*_*am*_), the residual variance ($\sigma _e^2$), the heritabilities of direct effects ($h_a^2$), maternal effects ($h_m^2$), the genetic correlation (*r*_*G*_), the heritability of the genetic component of the phenotype ($h_g^2$), and the heritability for the selection criterion in colony-based selection ($h_{CBS}^2$).

### Accuracy of breeding values

**Fig. 1:**
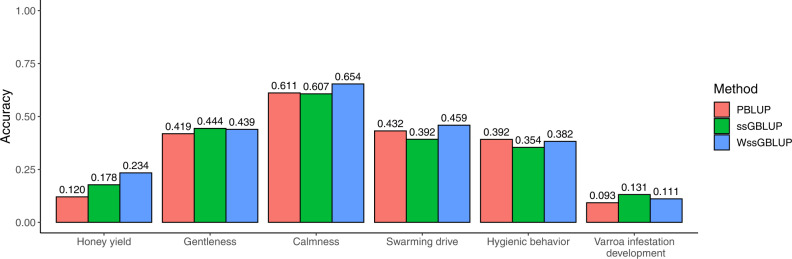
Accuracies of breeding values in the generation validation. Accuracies of pedigree-based BLUP (PBLUP), single-step genomic BLUP (ssGBLUP) and weighted ssGBLUP (WssGBLUP) were calculated in the generation validation.

The accuracies of the methods under investigation in the generation validation are shown in Fig. 1. Compared to PBLUP, the accuracy was improved with WssGBLUP for honey yield (94%), swarming drive (7%), gentleness (6%), calmness (5%), and VID (20%), and with ssGBLUP, improvements were observed for honey yield (48%), VID (41%), and gentleness (6%). The improvement with WssGBLUP over PBLUP for honey yield was statistically significant. No improvement was observed for hygienic behavior, and ssGBLUP did not yield a higher accuracy than PBLUP for calmness and swarming drive.

**Fig. 2:**
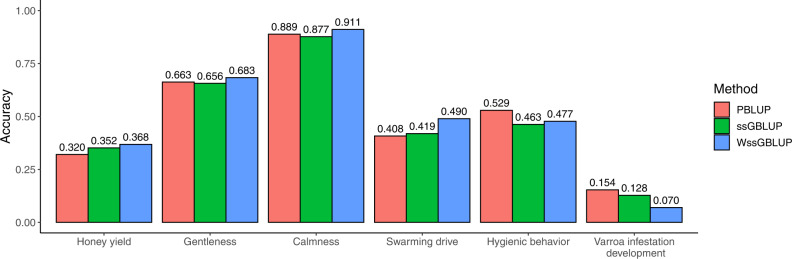
Mean accuracies in the five-fold cross-validation. Mean accuracies of pedigree-based BLUP (PBLUP), single-step genomic BLUP (ssGBLUP) and weighted ssGBLUP (WssGBLUP) were calculated in the five-fold cross-validation across the six repetitions. The standard errors over the six repetitions are not shown, as they were smaller than 0.02.

The accuracies of the methods under investigation in the five-fold cross-validation are shown in Fig. 2. Improvements over PBLUP were achieved for swarming drive (20%), honey yield (15%), calmness (2%), and gentleness (3%) with WssGBLUP. Improvement over PBLUP with ssGBLUP was achieved for honey yield (10%) and swarming drive (3%). The improvements with WssGBLUP over PBLUP were statistically significant for calmness and swarming drive. No improvement was observed for hygienic behavior and VID.

Overall, both validations showed similar results, although the accuracy was higher in the five-fold cross-validation, and the increases in accuracy with ssGBLUP and WssGBLUP over PBLUP were higher in the generation validation.

### Bias of breeding values

**Fig. 3:**
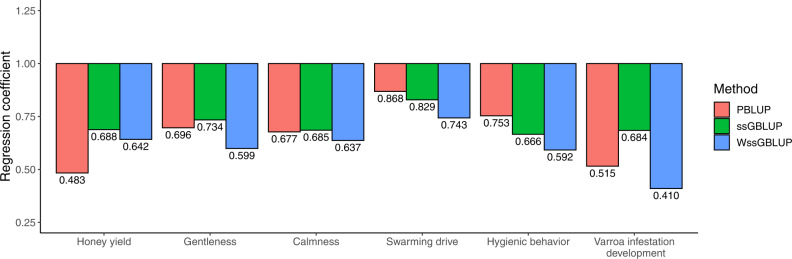
Bias of breeding values in the generation validation. Regression coefficients *b*_1_ of pedigree-based BLUP (PBLUP), single-step genomic BLUP (ssGBLUP) and weighted ssGBLUP (WssGBLUP) were calculated in the generation validation.

Bias was calculated as the regression coefficient *b*_1_ of phenotypes corrected by fixed effects on the predicted genetic component of the phenotype. The results for EBV from PBLUP, ssGBLUP and WssGBLUP in the generation validation are shown in Fig. 3. The results for all three methods showed inflated EBV estimates. The regression coefficient *b*_1_ deviated the most from 1 for VID with WssGBLUP and honey yield with PBLUP by −0.59, and −0.52, respectively. While WssGBLUP showed overall the most inflation, the difference between PBLUP and WssGBLUP ranged only up to 0.16, which was relatively small compared to the deviation from 1 with PBLUP. For ssGBLUP, the results were overall similar to PBLUP, although ssGBLUP was considerably less biased than PBLUP for honey yield and VID.

**Fig. 4:**
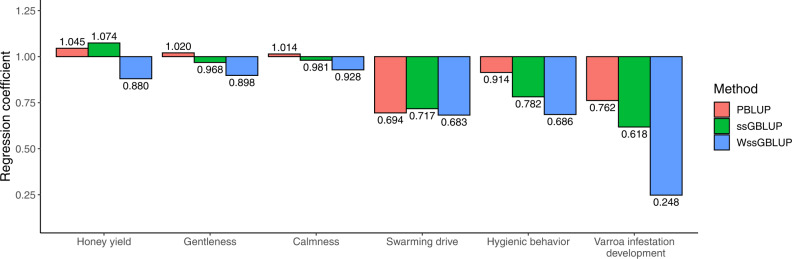
Bias of breeding values in the five-fold cross-validation. Mean regression coefficients *b*_1_ of pedigree-based BLUP (PBLUP), single-step genomic BLUP (ssGBLUP) and weighted ssGBLUP (WssGBLUP) were calculated in the five-fold cross-validation across the six repetitions. The standard errors over the six repetitions are not shown, as they were smaller than 0.04 for all other traits except for *Varroa* infestation development where they ranged up to 0.08.

The results for EBV from PBLUP, ssGBLUP and WssGBLUP in the five-fold cross-validation are shown in Fig. 4. For honey yield, gentleness, and calmness, the bias of the EBV was negligible, although the EBV from WssGBLUP tended towards inflation. For swarming drive and VID, all methods showed similarly inflated EBVs with regression coefficient *b*_1_ < 0.8. For hygienic behavior, EBVs from PBLUP were nearly unbiased, while the genomic methods produced inflated EBVs.

## Discussion

### Genetic parameters and quality of breeding values

The estimated heritabilities (Table 3) were in line with the results for the multiple trait models of the complete BeeBreed data set (Hoppe et al. 2020). The results on the accuracies in the generation validation (Fig. 1) and in the five-fold cross-validation (Fig. 2) showed improvements with WssGBLUP over PBLUP for honey yield, gentleness, calmness, and swarming drive. These results were within the range reported for data sets of similar size in dairy goats (Legarra et al. 2014), or for traits affected by maternal effects in beef cattle (Lourenco et al. 2015) or pigs (Putz et al. 2018).

The results on the difference in accuracy between WssGBLUP and PBLUP can be explained with the results on the heritabilities (Table 3). Traits with a higher heritability for maternal effects than for direct effects can be expected to show higher increases than other traits in accuracy with WssGBLUP and ssGBLUP over PBLUP, because simulation studies in honey bees showed greater increases in accuracy with ssGBLUP over PBLUP for maternal effects than for direct effects (Bernstein et al. 2021). This result stood out from other species where maternal effects are modelled, as in beef cattle (Lourenco et al. 2018) and simulation studies for beef cattle and pigs (Lourenco et al. 2013; Putz et al. 2018), the accuracy for direct effects showed higher increases in accuracy with ssGBLUP over PBLUP than the accuracy for maternal effects. The results of the current study are in line with the results from the simulations on honey bees (Bernstein et al. 2021). On the one hand, honey yield and swarming drive showed the highest improvements in accuracy with WssGBLUP over PBLUP, and the heritability for maternal effects is equal to or greater than the heritability for direct effects in both traits. On the other hand, gentleness, calmness, and hygienic behavior showed less or even no improvements in accuracy with WssGBLUP over PBLUP, and the heritability for direct effects is twice as great as the heritability for maternal effects in these traits.

The results for the *Varroa* resistance-related traits were also affected by problems in gathering data. The number of genotyped queens with phenotype for both traits was about 200 queens lower than for honey yield, gentleness, and calmness. Furthermore, the number of phenotyped queens on apiaries with a genotyped queen (Table 2) was low for the *Varroa*-related traits, which might have led to less accurate fixed effects. The results for VID are also due to the low heritability of the genetic component of the phenotype for this trait, because simulation studies in honey bees and other species show that traits with low heritability also have low accuracy of pedigree-based and genomic EBV (Gowane et al. 2019; Gupta et al. 2013). However, *Varroa*-specific hygienic behavior is the subject of ongoing research (Conlon et al. 2019; Farajzadeh et al. in prep; Mondet et al. 2020). The discovery of new quantitative trait loci (QTL) which are then covered by causative SNPs on a new chip can increase accuracy for the *Varroa*-related traits.

The accuracy of ssGBLUP was slightly lower than the accuracy of WssGBLUP for most traits. This result is common in studies for several other agricultural species using WssGBLUP (e.g., Lu et al. 2020; Teissier et al. 2019; Wang et al. 2014). In simulation studies (Lourenco et al. 2017; Wang et al. 2012), WssGBLUP had higher accuracy than ssGBLUP when the trait was controlled by few QTL, and both methods showed equal accuracy when the trait was polygenic. As the accuracy for VID was higher with ssGBLUP than with WssGBLUP in both validations, the genetic architecture of the trait appears to be highly polygenic. However, this is a preliminary conclusion, as VID has the lowest heritability of the traits we considered, due to the many factors that affect it (see Guichard et al. 2020 for a review).

The accuracies in the five-fold cross-validation were for the majority of the traits higher than in the generation validation. This is due to the fact that in the five-fold cross-validation, sibling groups are evenly distributed across the partitions, while the phenotypes of whole sibling groups might be removed for the calculation of EBVs in the generation validation. Therefore, the five-fold cross-validation is a validation within sibling groups, while the generation validation is similar to a validation across sibling groups. Studies in other species found that validations within sibling groups show higher accuracies than validations across sibling groups (Gao et al. 2019; Kjetså et al. 2020; Legarra et al. 2008). The standard errors of the accuracies in the five-fold cross-validation were extremely small in our study, but the accuracies for individual partitions showed large differences. This suggests that the predicted breeding values were stable across the repetitions, although the results on single partitions were very different.

According to a simulation study in honey bees (Bernstein et al. 2021), the size of the reference population in our study is close to the minimal size which should be available to initiate a breeding program. We expect the reference population to grow in the future, when breeders start to apply genomic selection.

The larger reference population is likely to obviate the need to run WssGBLUP instead of ssGBLUP, since a simulation study showed that WssGBLUP and ssGBLUP yield the same results for large reference sets (Lourenco et al. 2017). The larger reference population will also result in an increase of the accuracy of genomic methods, as results from other species demonstrate (Daetwyler et al. 2012; Lourenco et al. 2015; Mehrban et al. 2017; Moser et al. 2009).

In the generation validation, inflation was observed with all methods (Fig. 3). However, considerable bias was neither observed in simulations for honey bees (Bernstein et al. 2021) for PBLUB and ssGBLUP, nor the Austrian data set (Brascamp et al. 2016) with PBLUP. Since only limited bias was observed in the five-fold cross-validation (Fig. 4), the inflation in the generation validation is possibly due to genotype by environment interactions (GxE). GxE were found, e.g., in Italian honey bees (Costa et al. 2012), an Austrian honey bee breeding program (Brascamp et al. 2022), and by a wider study across Europe (see Meixner et al. 2014 for an overview). The five-fold cross-validation was less susceptible to GxE, since this validation only masked the phenotypes of one-fifth of the colonies on an apiary. Further analysis is required to confirm that the bias in the present study is due to GxE, and localize regions of similar GxE. The bias with genomic methods compared to PBLUP can be reduced by, e.g., increasing the share of the classic relationship matrix **A**_*g*_ in Eq. (9) (McMillan and Swan 2017; Misztal et al. 2017).

### Practical application of genomic selection in the honey bee

The availability of genomic breeding values offers new possibilities in breeding schemes for honey bees. In classical breeding schemes, queens spend the first months of their life building a colony. When the queens are 1 year old, they are used as drone-producing queens to inseminate other virgin queens, or phenotyped to be selected as dams of new queens when they are 2 years old. A simulation study of innovative genomic breeding schemes (Bernstein et al. 2021) suggested to genotype drone-producing queens before they are employed, and to employ only the candidates with the highest genomic breeding values. This requires additionally that phenotyped queens are genotyped to achieve a high accuracy of selection. According to the simulations, a budget to genotype at least 1000 queens per year should be available to increase genetic gain considerably. Another simulation (Brascamp et al. 2018) study argued for a different genomic breeding scheme, where several generations of queens are bred within a single summer by genomic selection, and phenotyped in the following year. Since this scheme implies a shorter generation interval, extremely high genetic gain would be possible, if the scheme was practically feasible.

Gathering genomic data from honey bees requires special considerations, due to their small body size, and their genetic diversity within a hive. Non-lethal ways to genotype queens are available (Jones et al. 2020), but require further development for commercial applications. The exuviae which queens leave behind after hatching offer a non-lethal option to genotype virgin queens, but just one exuvia is available for each queen, and exuviae showed low DNA quality in several cases. Relying purely on this technique in the present state could require breeders to forgo queens simply because the genotyping failed. Alternatively, drones can be gathered from a hive to genotype the queen, since drones are haploid offspring. However, collecting a sufficient number of drones in the first months after the queen’s hatching is impossible in routine breeding, since a young queen will only lay worker eggs to grow her colony.

## Conclusions

WssGBLUP offers significantly greater accuracy than PBLUP for honey yield, calmness, and swarming drive. For gentleness, the accuracy of WssGBLUP was greater than the accuracy of PBLUP to a similar degree as for calmness, but the difference remained below the threshold for significance. For all traits, except the *Varroa* resistance traits, the bias with WssGBLUP and ssGBLUP was on a similar level compared to the bias with PBLUP. For the *Varroa* resistance traits, the genomic methods offer too little improvement over PBLUP to be recommended based on the current data set, which is likely due to the size of the reference population. A larger reference population or the discovery of new causative SNPs for *Varroa* resistance are required to increase the accuracy of genomic methods for hygienic behavior and VID. The results suggest that genomic selection can be successfully applied to honey bees.

## Supplementary information

Approximation of the prediction accuracy for the genetic component of the phenotype

## Data Availability

The genotypes used for this study are available in Jones et al. (2020) (10.5061/dryad.gxd2547gp). The phenotype data of this study belong to several breeding associations and are unavailable due to legal reasons. Requests to access further raw material should be directed at the authors of this study.
