# Supplementary material for: First large-scale genomic prediction in the honey bee
Source: Heredity (Edinb). 2023 Mar 6;130(5):320–8. doi: 10.1038/s41437-023-00606-9 (PMC10163272; doi:10.1038/s41437-023-00606-9)
Supplement: Supplementary file 1 — Approximation of the prediction accuracy for the genetic component of the phenotype [file 41437_2023_606_MOESM1_ESM.docx]

**Text S1: Approximation of the prediction accuracy**

**for the genetic component of the phenotype**

In this appendix, we prove formula (14), which is used to estimate the accuracy of prediction from the correlation of estimated breeding values and phenotypes corrected by fixed effects. The phenotype corrected by fixed effect equals the breeding value plus the residual, which means:

|  | $r_{\boldsymbol{y}-\boldsymbol{Xb}\boldsymbol{,}\hat{\boldsymbol{g}}}=Corr\left( a_{W}+m_{Q}+e , \hat{a}_{W}+\hat{m}_{Q} \right) .$ | (A1) |
| --- | --- | --- |

Since the residual is independent of the estimated breeding values, we arrive at:

|  | $r_{\boldsymbol{y-Xb},\hat{g}}=\frac{\mathrm{Cov}(a_{W}+m_{Q},\hat{a}_{W}+\hat{m}_{Q})}{\sigma_{ph}\sqrt{\mathrm{Var}(\hat{a}_{W}+\hat{m}_{Q})}}\boldsymbol{.}$ | (A2) |
| --- | --- | --- |

The following formula is implied by Brascamp and Bijma (2019) in the paragraph leading up to (6a) in the same publication:

|  | $\mathrm{Var}\left( a_{W}+m_{Q} \right)=A_{base}\sigma_{a}^{2}+\sigma_{am}+\sigma_{m}^{2}.$ | (A3) |
| --- | --- | --- |

The definition of $h_{g}^{2}$ yields:

|  | $\frac{r_{\boldsymbol{y}-\boldsymbol{Xb}\boldsymbol{,}\hat{\boldsymbol{g}}}}{h_{g}}=\frac{\mathrm{Cov}(a_{W}+m_{Q},\hat{a}_{W}+\hat{m}_{Q})}{\sqrt{\mathrm{Var}(a_{W}+m_{Q})\mathrm{Var}(\hat{a}_{W}+\hat{m}_{Q})}}\boldsymbol{.}$ | (A4) |
| --- | --- | --- |

QED
